# Can a behaviour change toothbrushing intervention prevent dental caries in 11–13-year-olds?

**DOI:** 10.1038/s41432-024-01066-8

**Published:** 2024-09-18

**Authors:** Darshini Ramasubbu, Jonathan Lewney

**Affiliations:** 1 Dublin Dental University Hospital, Trinity College Dublin, Dublin, Ireland; 2 Independent Researcher and UK Specialist in Dental Public Health, Paris, France

**Keywords:** Dental public health, Preventive dentistry

## Abstract

**Design:**

The Brushing RemInder 4 Good oral HealTh (BRIGHT) multi-centre randomized controlled trial was based in state-funded secondary schools in England, Wales and Scotland. It had two arms, aiming to assess the clinical and cost effectiveness of a 50 min education session and twice daily brush reminder text messages on toothbrushing and caries rates, compared to the normal education curriculum and no SMS. Outcomes were assessed at intervals over 2.5 years and were assessor-blinded.

**Case selection:**

Pupils aged 11-13 were recruited from participating schools, and in each school randomised via year group to either the intervention or control group following baseline assessments by calibrated dental professionals. Exclusion criteria included not having a functioning mobile phone.

**Data analysis:**

The primary outcome, D_4–6_ MFT (Decayed, Missing and Filled Teeth), was analysed using mixed-effect logistic regression and sensitivity analyses were conducted. A cost-utility analysis was also undertaken.

**Results:**

In total 42 schools, containing 84 year groups were randomised, and 4680 pupils were in the final sample. 663 pupils withdrew from follow up. At 6 months, there was evidence that intervention group pupils were more likely to report brushing at least twice per day However, by 2.5 years this effect was no longer evident. 2383 participants had a valid dental assessment at both baseline and 2.5 years, with 514 children in the intervention and 529 children in the control group presenting with obvious decay experience in at least one permanent tooth after 2.5 years. The intervention was estimated to have a 7% chance of being cost-effective. Over the 2.5-year follow-up, there were no significant differences in QALYs and costs between groups.

**Conclusions:**

The findings from the BRIGHT trial indicate no evidence of a statistically significant difference between the intervention and control groups for the prevalence of caries after 2.5-years. The behaviour change intervention did not translate into a reduction in caries rates.

## A Commentary on

**Innes N, Fairhurst C, Whiteside K** **et al***.*

Behaviour change intervention for toothbrushing (lesson and text messages) to prevent dental caries in secondary school pupils: The BRIGHT randomized control trial. *Community Dent Oral Epidemiol* 2024; 10.1111/cdoe.12940.

**GRADE Rating:** Medium

## Commentary

This mobile health intervention focused on trialling the effectiveness of an education session and text message reminders on toothbrushing, analysing the subsequent impact on dental caries^1^. Interventions which target toothbrushing with a fluoridated toothpaste are popular due to their convenience, acceptability and effect on caries prevalence. This study adopted a solely behaviour change approach (aimed at increasing brushing at home), which was not found to be effective in reducing dental decay^1,2^.

This study demonstrated the ineffectiveness of and oral health promotion intervention in reducing dental caries in secondary school children^1^. Even when combined with daily text message toothbrushing reminders, no significant differences were found between the intervention and control groups^1^. This could be considered alongside Public Health England’s conclusion that ‘one off dental health education by dental workforce targeting the general population’ showed ‘evidence of ineffectiveness’ in preschool and school children^3^.

This well-conducted study focused on secondary school children, followed up for a longer time period compared to other mobile health interventions^1^. Randomised controlled trials measure the effectiveness of interventions prospectively, and use randomization to reduce bias and are therefore useful when investigating causality^4^. This study had a large sample size, with over 2300 children recruited from state schools from regions of England, Wales and Scotland attending both the baseline and final assessments^1^. Children had to have a mobile phone to participate, which may have excluded low income families^1^. The COVID 19 pandemic affected the timing of the final follow up and likely contributed to the attrition rate, though the authors stated the attrition rate was unlikely to have impacted the findings^1^.

The initial findings from the first 6 months indicated the intervention group were brushing more, though this was self-reported and not verified with more accurate measures, such as with haptic toothbrushes, as acknowledged by the authors^1^. In terms of the primary outcome, there was no statistically significant difference in caries between the groups after 2.5 years, which indicates that the combined education and SMS intervention was not successful and that resources may be best spent on alternative strategies to increase toothbrushing in this population^1^. Secondary outcomes, such as plaque score, were also similar between the two groups and, overall, the intervention was not found to be cost-effective^1^. The reporting of results such as these, which show evidence of ineffectiveness, are crucial in the allocation of limited resources. This was therefore a very useful trial to carry out and the results should be used to inform policy and practice.

Whilst further research is needed to identify effective and cost-effective measures to reduce caries in this population of secondary school children, recommended options currently include supervised tooth brushing in targeted settings such as schools, and the targeted provision of toothbrushes and toothpaste^3^.

Practice points

- A combined education and text message intervention did not prevent decay in secondary school children.
- Other interventions such as supervised toothbrushing with fluoridated toothpaste should be considered and may have a greater impact on dental caries, and be more cost-effective.
